# Relationship between Post-kidney Transplantation Antithymocyte Globulin Therapy and Wound Healing Complications

**Published:** 2012-05-01

**Authors:** G. R. Pourmand, S. Dehghani, A. Saraji, S. Khaki, S. H. Mortazavi, A. Mehrsai, H. Sajadi

**Affiliations:** 1 *Urology Research Center, Sina Hospital, Tehran University of Medical Sciences, Tehran, Iran*; 2 *Students’ Scientific Research Center, School of Medicine, Tehran University of Medical Sciences, Tehran, Iran*

**Keywords:** Kidney transplantation, Antithymocyte globulin, Wound healing complication

## Abstract

**Background: **Wound healing disorders are probably the most common post-transplantation surgical complications. It is thought that wound healing disturbance occurs due to antiproliferative effects of immunosuppressive drugs. On the other hand, success of transplantation is dependent on immunosuppressive therapies. Antihuman thymocyte globulin (ATG) has been widely used as induction therapy but the impact of this treatment on wound healing is not fully understood.

**Objective: **To investigate wound healing complications after ATG therapy in renal transplant recipients.

**Methods: **The medical records of 333 kidney transplant recipients were assessed for wound healing disorders. Among these patients, 92 received ATG and 5 doses of 1.5 mg/kg ATG along with the standard protocol of drugs.

**Results: **The mean age of patients was 38.9 years. Of 333 recipients, 92 (23.7%) received ATG; 21 (6.3%) developed wound healing complications. There was a significant relationship between ATG therapy and wound complications (p=0.034). Also, women were more likely to develop wound healing disorders than men (p=0.002). No statistical difference was observed between age and wound healing complication (p=0.28). There was no significant difference between the mean duration of hospitalization between ATG and Non-ATG group (p=0.9).

**Conclusion:** ATG increases the risk of overall wound complications. It is needed to pay more attention to the patients treated with this immunosuppressant to avoid the risk of re-interventions, lessen the duration of hospitalization and decrease the impairment of graft function.

## INTRODUCTION

There are several surgical complications occurring after kidney transplantation. Arterial and venous thrombosis [1], and ureteral leaks and strictures [2,3] are some of complications. The important and dangerous complications are generally uncommon but may occur in less than 5% of the patients [4]. At present, wound healing complications are probably one of the most common post-transplantation surgical complications [4]. Wound healing disorders have been reported as serious post-operative problems which occur approximately in up to 20% of all kidney transplant recipients [5]. Wound complications are not life- or graft-threatening but can lead to significant morbidity, prolonged hospitalization, hospital re-admission, and re-operation, increasing the overall cost of transplantation [4,6-9]. Surgical site infection, a frequent complication of kidney transplantation, is reported to occur in 4%–7.5% of patients with higher incidence in obese patients. It is associated with substantial morbidity and also lower patient and graft survival [10].

It is thought that wound healing disturbance occurs due to antiproliferative effects of drugs. Some comorbidities and immunosuppressive drugs may also impair wound healing. Other factors such as smoking and high body mass index (BMI) influence wound healing as well [4,11-17]. The rate of lymph leakage, lymphocele, wound dehiscence and hernia is high following immunosuppressive therapy in kidney transplant recipients [3]. The success of transplantation is however, dependent on immunosuppressive therapy. Induction therapy has become a part of standard care in many transplantation centers which results in reduced acute rejection after transplantation. Over the last decade, the use of induction therapy has increased from 30% to 75% [18]. Multiple agents have demonstrated beneficial effects for induction therapy including rabbit antithymocyte globulin (rATG), thymoglobulin, genzyme, alemtuzumab, basiliximab and daclizumab [19,20]. Thymoglobulin is a rabbit antihuman thymocyte globulin (rATG) that has been widely used as induction therapy for transplant recipients who are at high risk of rejection or delayed graft function. The impact of this induction therapy on wound healing is not fully understood [21].

To the best of our knowledge, only few large cohort studies on kidney transplant patients have been conducted to assess the impact of ATG induction therapy on wound healing. The present study investigates wound healing in ATG treated kidney transplant recipients.

## MATERIALS AND METHODS

In this study, the rate of wound healing complications among 333 kidney transplant recipients was determined from September 1994 to February 2010. Institutional Review Board approval was granted by the Research Ethics Committee of Tehran University of Medical Sciences. The standard protocol of drugs administered included 500 mg methylprednisolone (for induction) before the surgery, the first and the second post-operative day, then reduced to 1 mg/kg from the third day. For 241 of patients, ATG was not part of our routine protocol (non-ATG group). Patients in ATG group received 5 doses of 1.5 mg/kg ATG with a dose reduction for leukopenia and/or thrombocytopenia. Mycophenolate mofetil (MMF; Cellcept) was prescribed at the dose of 1 g/BD; if patients did not receive ATG, it was started from the fifth day of the surgery. Cyclosporine (3 mg/kg in ATG group and 6 mg/kg in non-ATG group) was added simultaneously to the protocol once the serum creatinine fell below 2.5 mg/dL. Patients were hospitalized for at least one week and those who received at least 72 hours of ATG within six weeks of transplantation were included in the study. 

The medical records of both ATG and non-ATG groups were assessed for wound dehiscence, lymphocele, urine leak, hematoma and wound discharge. Other variables including demographics information, donor source, date of transplantation, cause of transplantation, premorbidity, pre- and post-operative therapy, rejection, duration of hospitalization and presence and type of complications were also investigated.

Statistical analysis was performed by SPSS^®^ ver 17. Statistical significance was considered as a value of p<0.05.

## RESULTS

In this study, medical records of 333 patients who underwent kidney transplantation from September 1994 to February 2010 were reviewed. Among these patients, 61% were male and 39% were female. The mean age of recipients was 38.9 years (39.5 for men and 37.8 for women). Table 1 shows other characteristics of participants. In most patients (33.9%) the cause of end-stage renal disease (ESRD) was unknown (Table 2). Of 333 recipients, 92 (23.7%) received ATG. Table 3 shows some studied variables in patients of ATG and non-ATG groups. Twenty-one (6.3%) patients developed wound healing complications including lymphocele (n=9), urine leak (n=1), wound dehiscence (n=6), hematoma (n=1) and wound discharge (n=4). Among these patients, 10 (10.8%) were in ATG group and 11 (4.5%) were in non-ATG group (Table 4). There was a significant relationship between ATG therapy and wound complications (p=0.034). As shown in Table 5, six of the patients who developed wound healing complications were male (28.6%) and 15 were female (71.4%) (p=0.002).

**Table 1:** Characteristics of participants

Variable	Value
Patient age (yrs), mean (range)	38.9 (9–67)
Sex, M/F, n (%)	203 (61%)/130 (39%)
Body mass index (kg/m^2^), mean (range)	22.3 (13.8–31.9)
Previous transplantation, n(%)	12 (3.6%)
Pre-Tx* dialysis (month), mean (range)	19.1 (0–130)
Donor, Living R†/Living UnR‡/CAD§	15 (4.5%)/289 (86.8%)/29 (8.7%)
Hospitalization (day), mean (range)	22 (7–120)

* Transplant,

† Related,

‡ Unrelated,

§ Coronary artery diseases

**Table 2:** Cause of ESRD in participants

Cause	n (%)
Unknown	113 (33.9)
Hypertension	104 (31.2)
Diabetes mellitus	40 (12.0)
Glomerulonephritis	21 (6.3)
Polycystic kidney disease	11 (3.3)
Stone	11 (3.3)
Others	32 (9.6)

**Table 3:** Characteristics of ATG and non-ATG treated renal transplant recipients

Variable	ATG (Cases)	Non-ATG (Controls)
Patient age, mean (y)	39.4	38.6
Male (%)	62.7	56.5
Donor (%)		
Living related (%)	4.3	4.6
Living unrelated (%)	68.5	93.8
Cadaveric (%)	27.2	1.7
DM* as the cause of ESRD (%)	14.1	11.2
Hospitalization, mean (day)	26.9	20.1

* Diabetes mellitus

**Table 4:** Wound healing complications distribution by ATG therapy

	ATG therapy			p value
Complication	Yes	No	Total	
Dehiscence	3	3	6	
Lymphocele	3	6	9	
Urine leak	1	0	1	
Hematoma	1	0	1	
Wound discharge	2	2	4	
Total	10 (10.8%)	11 (4.5%)	21 (6.3%)	0.034
Total Patients	92	241	333	

**Table 5:** Wound healing complication distribution by gender

	Gender			p value
Complication	Male	Female	Total	
Dehiscence	1	5	6	
Lymphocele	4	5	9	
Urine leak	0	1	1	
Hematoma	1	0	1	
Wound discharge	0	4	4	
Total	6 (2.9%)	15 (11.5%)	21 (6.3%)	0.002
Total Patients	203	130	333	

The mean age of patients with would healing complications was 39.2 (range: 20–67) years while it was 38.8 (range: 9–65) years for the those who did not develop wound healing disorders (p=0.28). The mean duration of hospitalization (Table 1) after the surgery was 21.4 days in patients with complete wound healing and 30.6 days in patients with wound healing complications (p=0.9). Patients underwent dialysis 19.1 months in average before the transplantation; 39 (11.7%) patients had no experience of dialysis.

## DISCUSSION

Iran has the highest number of renal transplant recipients in the Middle East [22]. Up to 2006, 21,359 kidneys have been transplanted in our country [23]. Annually, 1500 to 1700 recipients are added to this population [22]. Nowadays, renal transplantation is routinely performed around the world and complications are the most troublesome issue in this field. At present, wound healing complications are probably one of the most common post-transplantation surgical complications. Different factors have been investigated in various studies as the responsible factors for wound healing complications such as immunosupressant agents, steroids, elevated BMI, smoking and rejection episodes. For the drugs as a major risk factor, it is thought that wound healing disturbance occurs due to antiproliferative effects of drugs which impair fibroblasts function [3-5,13,14,17,24-26].

In the present study, 6.3% of patients developed wound healing complications (10.8% in ATG *vs*. 4.5% in non-ATG group); among them, lymphocele was the most frequent complications recorded. Grim, *et al* [14], determined the incidence of surgical site complications among renal transplant recipients who received sirolimus with MMF. They reported an incidence of 31.8% for wound healing complications with the highest incidence for wound dehiscence. Flechner, *et al* [13], reported a rate of 16.2% for wound healing complications.

In the study of Benavides, *et al* [21], the percentage of wound complications in a group receiving rATG for induction for a maximum of two weeks post-operatively was 39.1% compared with 26.0% for patients who received basiliximab induction (an overall incidence of 30.6%); they found a significant difference between these two groups (p=0.025). Our findings are in good agreement with the results of Benavides, *et al*. In the current research, there was a significant difference in the rate of wound complications between ATG and non-ATG groups (p=0.034) but in the report of Grim, *et al* [14], although the incidence of complications was lower in the control group, they found no statistically significant differences (p=0.163). The highest complication rates in Benavides, *et al*, study was incisional hernia in 13.1% followed by wound infection in 12.9% and lymphocele requiring drainage in 12.0% of recipients.

In our study 11.5% of women compared with 2.9% of men developed wound healing complications (p=0.002). This finding was in keeping with the finding of Benavides, *et al* [21], who reported that 51.4% of women were in group with complication compared with 37.4% in the group with no complication (p<0.025). They suggested that gender is a risk factor for developing wound complications. They could explain this difference by presenting more women receiving rATG in group with complication compared with those without wound complications (45.5% *vs*. 29.7%). But we cannot justify the difference in this way. In the present study, the percentage of women in ATG group was 37.3% compared with 47.5% in non-ATG group. Benavides, *et al* [21], found a significant relationship between metabolic disorders and wound complications. They found that 35.6% of patients with post-operative wound infection had diabetes mellitus whereas only 21.3% of patients without infection had this metabolic disorder. Grim, *et al* [14], also observed such relationship in their study. The rate of diabetes mellitus was higher among patients receiving sirolimus and who had complications compared with the control group, though the difference was not significant. Aneesh Srivastava, *et al* [27], compared two groups of patients who received MMF and Sirolimus. They found wound infection in 7.5% and 5% (p=0.646) and wound dehiscence in 2.5% and 20% (p=0.014) of the groups, respectively.

In our study, the mean duration of hospitalization after the surgery was 21.4 days in patients with complete wound healing and 30.6 days in patients with wound healing complications (p=0.9). However, Aneesh Srivastava, *et al* [27], found that the duration of hospitalization was significantly higher (35 *vs*. 24 days) in patients who received Sirolimus, because they had more frequently developed wound healing disorders. On the contrary, Kai Lopau, *et al* [5], compared two groups of patients who received MMF and azathioprine. Wound healing disturbance was observed more often in the azathioprine group (17% *vs*. 10%), though this difference was not statistically significant (p=0.24).

We concluded that ATG increases the risk of overall wound complications. It is needed to pay more attention to the patients treated with this immunosuppressant to avoid the risk of re-interventions, lessen the duration of hospitalization and decrease the impairment of graft function.
